# PPARα in the Epigenetic Driver Seat of NAFLD: New Therapeutic Opportunities for Epigenetic Drugs?

**DOI:** 10.3390/biomedicines10123041

**Published:** 2022-11-25

**Authors:** Claudia Theys, Dorien Lauwers, Claudina Perez-Novo, Wim Vanden Berghe

**Affiliations:** Lab Protein Chemistry, Proteomics & Epigenetic Signaling (PPES), Department of Biomedical Sciences, University of Antwerp, 2610 Wilrijk, Belgium

**Keywords:** NAFLD, epigenetics, metabolism, nuclear receptor, PPARα

## Abstract

Nonalcoholic fatty liver disease (NAFLD) is a growing epidemic and the most common cause of chronic liver disease worldwide. It consists of a spectrum of liver disorders ranging from simple steatosis to NASH which predisposes patients to further fibrosis, cirrhosis and even hepatocarcinoma. Despite much research, an approved treatment is still lacking. Finding new therapeutic targets has therefore been a main priority. Known as a main regulator of the lipid metabolism and highly expressed in the liver, the nuclear receptor peroxisome proliferator-activated receptor-α (PPARα) has been identified as an attractive therapeutic target. Since its expression is silenced by DNA hypermethylation in NAFLD patients, many research strategies have aimed to restore the expression of PPARα and its target genes involved in lipid metabolism. Although previously tested PPARα agonists did not ameliorate the disease, current research has shown that PPARα also interacts and regulates epigenetic DNMT1, JMJD3, TET and SIRT1 enzymes. Moreover, there is a growing body of evidence suggesting the orchestrating role of epigenetics in the development and progression of NAFLD. Therefore, current therapeutic strategies are shifting more towards epigenetic drugs. This review provides a concise overview of the epigenetic regulation of NAFLD with a focus on PPARα regulation and highlights recently identified epigenetic interaction partners of PPARα.

## 1. Introduction

Nonalcoholic fatty liver disease (NAFLD) is a growing epidemic mirroring the increase in obesity and diabetes mellitus in Western diet-consuming countries. The estimated prevalence of NAFLD is currently 20–30% in Europe. Moreover, it is the most common cause of chronic liver disease worldwide [1,2]. NALFD consists of a spectrum of liver disorders, ranging from isolated steatosis (nonalcoholic fatty liver, NAFL) to nonalcoholic steatohepatitis (NASH) and fibrosis. The majority of patients have isolated steatosis, which is often considered benign in nature, whereas NASH can lead to complications such as fibrosis, cirrhosis and hepatocellular carcinoma (HCC), as well as extrahepatic diseases, especially cardiovascular disease [3,4,5]. Considering the risk for further complications, patients are often clinically stratified in non-NASH or NASH. However, current knowledge shows a higher complexity and spectrum in disease phenotypes, causes and progression. Therefore, recently it has been suggested that the name of NAFLD is revised to metabolic-associated fatty liver disease (MALFD). This more generic term allows to stratify patients into subphenotypes reflecting the dominant driver of the disease based on genetic, anthropometric and metabolic phenotyping approaches, allowing individualized risk prediction and prevention strategies [6]. Moreover, this gives an opportunity for better clinical trial design, leading to better knowledge of the mechanism behind the pathological progression of NAFLD, which is crucial, but incomplete at present.

Today, it is generally accepted that the interplay between environmental factors, genetics and epigenetics plays a crucial role in the development of NAFLD. More specifically, a lipid-rich diet and a lack of exercise can be linked to the development of NAFLD [7]. Therefore, the only treatment for NAFLD that is currently recommended consists of a change in lifestyle, including a change in diet and a lot of exercise. However, this is difficult to maintain for patients and therefore many of them relapse [8]. Moreover, NAFLD has also been diagnosed in lean patients without obesity or diabetes, for whom this treatment is less appropriate [9]. Furthermore, genetic mutations have also been linked to the development of NAFLD. For example, mutations in the patatin-like phospholipase domain-containing 3 (PNPLA3) gene are considered a hallmark for the development of NAFLD [10,11]. However, both environmental factors and genetics cannot fully explain the high prevalence of NAFLD. Therefore, researchers are also searching for epigenetic factors contributing to the development and progression of the disease. Although it is known that NAFLD is linked to a global DNA hypomethylation and a hypermethylation of peroxisome proliferator-activated receptor-α (PPARα) gene (promoter) sequences, little is known about the key players and mechanism behind this epigenetic regulation [12,13]. Therefore current drug research is mainly focused on agents targeting lipid metabolism, inflammatory or fibrotic pathways, i.e., lipid-lowering agents (e.g., statins), antioxidants (e.g., vitamin E) and agents activating key players in lipid metabolism (e.g., PPARα, sirtuin 1 (SIRT1), AMP-activated protein kinase (AMPK)) [14].

PPARα is a nuclear receptor which is part of the PPAR family consisting of three members: PPARα, PPARβ/δ and PPARγ. These three receptors are expressed from different genes and each isotype is highly expressed in different tissue [15,16]. PPARα is largely expressed in the liver and brown adipose tissue, followed by the heart and the kidneys. PPARβ/δ is ubiquitously expressed in tissues with high peroxisomal and mitochondrial β-oxidative activity, including skeletal muscle. PPARγ is mainly expressed in white adipose tissue [16]. Since PPARα is highly expressed in the liver and is a key regulator of the lipid metabolism, a lot of research has focused on this receptor in the treatment of NAFLD. This research has already shown that PPARα is a key player in the pathogenesis of NAFLD, since it is hypermethylated and downregulated in NAFLD patients [13,17]. However, agonists targeting PPARα specifically (e.g., fibrates), have not shown promising results in clinical trials [18].

Since various PPARα target genes related to lipid metabolism have shown strong DNA methylation variation in NAFLD, research has shifted towards the identification of key players in this epigenetic regulation. Interestingly, several recent articles have shown a direct interaction of PPARα with epigenetic enzymes affecting the lipid metabolism. Therefore, we have summarized the epigenetic regulation of PPARα in NAFLD, as well as PPARα-interacting epigenetic enzymes which may represent interesting targets for future treatment options.

## 2. The Peroxisome Proliferator Activated Receptor Alpha—PPARα

### Structure and Regulation of PPARα

**Figure 1:**
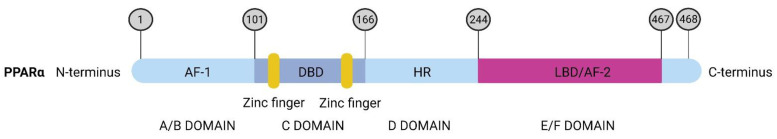
Schematic view of the protein structure of PPARα and domain function. First there is the A/B domain or the activation function one (AF-1) domain which works without ligand binding. Next there is the C-domain or DNA-binding domain (DBD) containing two highly conserved zinc finger-like motifs shown in yellow. Following is the hinge region connecting the C domain with the last E/F domain, known as the ligand-binding domain (LBD) with the activation function two (AF-2).

The *PPARA* gene consists of eight exons and it is mapped to chromosome 22 in humans and chromosome 15 in the mouse. It encodes for the PPARα protein which is 468 amino acid residues long (Figure 1). This protein contains five functional domains, from A to F. First, at the N-amino terminal there is the A/B domain or the activation function one (AF-1) domain. This domain works independently without the binding of a ligand. Second, next to the AF-1 domain there is the C-domain or DNA-binding domain (DBD) containing two highly conserved zinc finger-like motifs. The binding of the receptor to the peroxisome proliferator response element (PPRE) sequence of target genes is promoted by these zinc finger-like motifs. Third is the D-domain or hinge region (HR) that connects the C-domain with the E/F domain. Last, at the C-terminus there is the E/F domain or ligand-binding domain (LBD) with the activation function two (AF-2). Ligands can bind to the LBD leading to the stabilization and recruitment of cofactors by AF-2. The co-regulators can bind to PPARα with their LXXLL domain [19].

Generally, the ligands of PPARα are divided into two main groups: one group of natural ligands and another of synthetic ligands. The natural ligands consist of endogenous (e.g., free fatty acids, derived from the lipid metabolism) and exogenous (e.g., resveratrol, derived from the diet or medicinal plants) molecules [20,21].

In the cell, the absence of PPARα ligands leads to the inactivation of the receptor by co-repressors. After ligand binding, the co-repressors will be replaced by co-activators resulting in the heterodimerization with retinoid X receptor (RXR). This complex can bind to specific PPREs resulting in the transcription of target genes [16,22]. Most of these target genes are involved in lipid metabolism or fatty acid (FA) catabolism, including genes involved in FA binding, transport, and degradation via mitochondrial or peroxisomal oxidation. Other pathways include ketogenesis, amino acid metabolism, glucose metabolism and inflammation [23].

## 3. Epigenetic Regulation of PPARα in NAFLD

### 3.1. Epigenetics

Epigenetics is the study of reversible changes in gene expression that can be inherited through cell division, but are not caused by DNA sequence alterations [24]. Epigenetic modifications consist of DNA methylation, histone modifications and microRNAs [25].

First, DNA methylation is known as the addition of a methyl group (-CH3) on the fifth carbon of the pyrimidine ring in cytosine, generating 5-methylcytosine (5meC). This process is managed by DNA methyltransferases (DNMTs) and is most often found in CpG islands of the promoter region. Hence, CpG island hypermethylation typically results in the inhibition of gene transcription. The family of DNMTs consists of three isoforms: DNMT1 which maintains the DNA methylation pattern during DNA replication, and DNMT3a and DNMT3b responsible for de novo methylation [26,27]. Since DNA methylation is a dynamic process depending on environmental cues and biological context, this methyl group can also be removed. The first step in active DNA demethylation consists of the hydroxylation of 5meC to 5-hydroxymethylcytosine (5hmC) mediated by DNA dioxygenases known as ten-eleven translocation (TET) enzymes. These enzymes are also responsible for the further sequential oxidation of 5hmC to 5-formylcytosine (5fC), and 5-carboxycytosine (5caC). Final DNA demethylation will then occur in a two-step manner. First, 5fC and 5caC will be excised by thymine-DNA-glycosylase (TDG), followed by a replacement with an unmodified cytosine due to the base excision repair mechanism [28,29]. The TET family consists of three members: TET1, TET2 and TET3. All TET proteins have the same catalytic activity but are expressed in different tissues and related to different biological processes. TET1 is highly expressed in embryonic stem cells (ESC) and primordial germ cells. TET2 is also expressed in ESC, while TET3 is expressed in oocytes, zygotes and neurons. Both TET1 and TET2 are important for the correct differentiation of ESC [28,30]. Moreover, TET2 is also important for the hematopoietic stem cell differentiation [31]. The TET3 protein is important for the complete erasure of 5mC of the paternal genome after fertilization and the correct neuronal differentiation [32,33]. Although the study of TET enzymes has mostly been performed in ESC, the correct expression of these enzymes in differentiated tissues has also been proven to be important. TET2 mutations have been associated with myeloid malignancies and aberrant expression due to changes in steroid hormone regulation, while the aberrant expression of TET1 has been related to a worse outcome of reproductive-related cancers [28,31,34].

Second, histone modifications consist of the post-translational acetylation (lysine), methylation (lysine/arginine) and phosphorylation (threonine/serine) of the N-terminal tail of the different histones H2A, H2B, H3 and H4 [26,27]. These modifications are catalyzed by histone-modifying enzymes that can be divided into three classes: writers, readers and erasers. Writers are enzymes that can add modifications to the histone tails including histone methyltransferases (HMTs; including lysine methyltransferases (KMTs), e.g., Enhancer of zeste homolog 2 (EZH2) and arginine histone methyltransferases (PRMTs), e.g., PRMT5), histone acetyltransferases (HATs) and ubiquitin ligases. These modifications can then be removed by erasers including lysine demethylases ((KDMs), e.g., jumonji D3 (JMJD3)), histone deacetylases (HDACs) and deubiquitinating enzymes [27,35,36]. Since histones are responsible for the conformation and stability of the DNA, specific combinations of these modifications promote the binding of specific protein complexes known as readers. Depending on the protein complexes, this will result in the activation or silencing of gene transcription [26,27].

Third, microRNAs (miRNAs) suppress mRNA translation by altering protein expression. MicroRNAs are endogenous, short (approximately 18–25 nucleotides), non-coding RNA molecules with an important post-transcriptional regulatory role. They target the 3′-untranslated region (3′UTR) of specific mRNA leading to inhibited translation or mRNA degradation [37]. The following section will discuss the epigenetic alterations in NAFLD with a focus on PPARα.

### 3.2. Methylation State of PPARα Is a Biomarker of NAFLD Development

Overall, NAFLD patients show aberrant DNA methylation levels (5meC) correlated with the severity of the disease. More specifically, compared to controls, a low hepatic global DNA methylation level is present in NAFLD patients which further decreases when mild inflammation and moderate fibrosis occur [12]. Moreover, NAFLD patients with mild versus severe fibrosis can be distinguished based on the lower methylation of specific CpGs in pro-fibrogenic genes in NAFLD patients with severe fibrosis [38]. Besides methylation, Pirola et al. reported that NAFLD patients also show a significant loss of non-nuclear hydroxymethylation (5hmC) based on immune-specific assays. This non-nuclear 5hmC is probably located in the mitochondria. Hepatic nuclear 5hmC in the livers of NAFLD patients is however not significantly altered compared to controls or different stages of the disease. Interestingly, a positive correlation of 5hmC with the mitochondrial DNA copy number and an inverse correlation with peroxisome proliferator-activated receptor-gamma coactivator 1α (PPARGC1α) mRNA levels have also been found [39]. This suggests that besides 5mC, 5hmC may also contribute to the pathogenesis of NAFLD by the regulation of mitochondrial biogenesis and PPARGC1A expression. Since PPARGC1α is a major modulator of mitochondrial biogenesis and NAFLD is associated with changes in PPARGC1α expression, mitochondrial function and copy number [39,40,41]. 

Further evidence of the crucial role of epigenetic regulation in the development of NAFLD can be found in rodent studies using different diets. DNA methylation can be influenced by diet nutrients such as choline, methionine and betaine. These components are considered “methyl donors” promoting DNA methylation [42,43]. Supplementation of these methyl-donors can lead to an increase in the hepatic outflow of triglycerides [44]. For example, betaine is a methyl donor generally existing in food, such as spinach and shrimps, that plays an important role in the prevention and therapy of liver diseases including NAFLD [45]. Interestingly, the DNA methylation pattern of PPARα can be modified by betaine resulting in improved triglyceride content [44,46,47]. Reciprocally, deficiency of methyl donors results in triglyceride accumulation by the overexpression of genes associated with fatty acid synthesis leading to a NAFLD-like situation [48]. Besides methyl donors, lipids and fructose also influence DNA methylation. For example, the offspring of female mice fed a high-fat diet (HFD) before and during gestation and lactation, followed by an HFD after weaning developed NAFLD with increased methylation of PPARα in offspring. Similarly, offspring of female rats put on a high fructose diet revealed increased methylation of key metabolic genes including PPARα [49,50]. Both studies indicated that a bad maternal environment can epigenetically predispose the offspring to metabolic diseases, including NAFLD [49,50]. Moreover, all the previous data indicate that the nuclear receptor PPARα a key factor is in the epigenetic regulation of NAFLD.

Interestingly, both altered DNA methylation and hydroxymethylation patterns have been observed at the PPARα gene locus in NAFLD conditions. More specifically, PPARα was hypermethylated in an in vitro and in vivo steatosis model leading to lower PPARα gene expression and protein levels [13]. This is similar to NAFLD patients showing gradually decreasing PPARα expression levels, with each advanced stage of NAFLD [17]. Besides methylation, hydroxymethylation has also been shown to influence PPARα expression in NAFLD. Wang et al. [51] proved that TET1 can directly bind to the promoter region of PPARα-mediating hydroxymethylation. This might suggest that TET1 has a protective effect against NAFLD by demethylating and thus increasing the hydroxymethylation of PPARα, promoting fatty acid oxidation. Moreover, TET1 knockout mice resulted in a higher degree of liver steatosis and lower levels of PPARα and its target genes [51].

Since the DNA hypermethylation of the PPARα gene is linked to the development of NAFLD, researchers have tried to alleviate NAFLD progression by inhibiting the DNA methylation of the PPARα gene by natural herbal compounds. For example, curcumin, a traditional Chinese and Indian medicine isolated from turmeric (Curcuma longa) was shown to reverse the NAFLD phenotype in vitro and in vivo by reducing the methylation of several genes including DNMT1 and PPARα, resulting in increased PPARα expression [13,52,53].

### 3.3. Histone Modifications at the Promoter Region of PPARα Related to the Development of NAFLD

Another layer of gene expression regulation by epigenetic modifications are histone modifications. These modifications can alter chromatin structure and thus the accessibility for transcription factors [26,27]. A growing body of literature has investigated histone methylation and acetylation in NAFLD leading to changes in PPARα expression.

Previous studies have shown that a deficiency in histone demethylase Jhdm2a (also known as Jumonji domain containing 1 (JMJD1A)) induces the development of the hallmarks of metabolic syndrome including hyperlipidemia and obesity. Jhdm2a is responsible for the demethylation of H3K9 and can thereby regulate the expression of multiple genes [54,55]. Interestingly, Tateishi et al., found that in skeletal muscle cells, this change in lipid metabolism was due to the direct binding of Jhdm2a to PPARα. More specifically, Jhdm2a knockout mice had an increased level of the inhibitory H3K9me2 modification at the promoter region of PPARα which triggered decreased PPARα expression and downstream PPARα target genes involved in lipid metabolism including fatty acid oxidation [55]. Moreover, hepatic transcriptome profiling of HFD-induced NAFLD mice revealed an altered expression of genes encoding jumonji C-domain-containing histone demethylases (JMJD) that can regulate histone trimethylation (e.g., H3K9me3 and H3K4me3) [56]. Accordingly, in lipid-accumulated hepatocytes, H3K9me3 and H3K4me3 levels diminished at the promoter region of PPARα and hepatic lipid catabolism gene networks resulting in their reduced expression [56]. Besides lysine methyltransferase, PRMT5 activity has also been associated with the inhibition of PPARα functions upon HFD [57]. PRMT5 is part of the arginine methyltransferase family (PRMT) consisting of three subfamilies which differ in their ability to carry out monomethylation, asymmetric demethylation (type I), monomethylation or symmetric demethylation (type II) or exclusively monomethylation (Type III) [58]. PRMT5 is a known type II arginine methyltransferase that dimethylates histones H2AR3 [59], H4R3 [60] and H3R8 [61] but also non-histone proteins including SREBP1 and AKT kinase [57,62]. Huang et al. showed that an HFD induces the activation of AKT kinase by PRMT5, which will further phosphorylate and inhibit PPARα functions. This will lead to an inhibition of mitochondrial β-oxidation and aggravation of a high-fat diet-induced hepatic steatosis [57]. All these studies indicate that the epigenetic regulation by histone methylation is a putative hallmark for the development of NAFLD and the regulation of PPARα.

Furthermore, increased histone acetylation levels have also been observed in an in vitro steatosis model and contribute to the development of NAFLD [63]. Accordingly, HDAC inhibitors such as sodium butyrate can alleviate HFD-induced NAFLD by increasing β-oxidation. This could be explained by restoring the acetylation pattern and expression of PPARα. More specifically, sodium butyrate enhances the H3K9Ac modification at the PPARα gene promoter [64].

Altogether, although data on histone modifications in metabolic diseases including NAFLD and key players such as PPARα remain fragmentary, the current data already highlight the importance of histone methylation and acetylation regulation of PPARα in the development of NAFLD. Future studies will need to further untangle the histone modification landscape of NAFLD.

### 3.4. PPARα-Targeting microRNAs Contribute to NAFLD Development

Previous studies have shown that several miRNAs are upregulated in NAFLD patients, as well as in experimental in vitro and in vivo NAFLD models [65]. Today, miRNAs are considered important post-transcriptional modulators in NAFLD pathology, which can mimic gene silencing. Some of these altered miRNAs target nuclear receptors, including PPARα [65]. For example, miR-200, miR-20b, miR181-a, miR-30a-3p, miR519d, miR-21 and miR-22 are elevated in NAFLD and directly target PPARα mRNA [66,67,68,69,70,71,72]. The working mechanism of these miRNAs leading to the aggravation of NAFLD is approximately the same. They all bind to the 3′UTR of PPARα mRNA resulting in PPARα mRNA degradation, decreased protein expression and disturbed lipid metabolism, leading to the aggravation of an NAFLD phenotype. Moreover, the induced expression of specific miRNAs (miR-20b, miR181-a, miR-30a-3p and miR-22) in FFA-treated hepatocytes increased the intracellular lipid content upon reduction in PPARα mRNA levels and decreased protein expression [66,67,69,70]. Moreover, even in colorectal cancer-derived liver metastasis, deregulated PPAR targeting miRNAs have been observed [73].

Therefore, antagomirs targeting specific miRNAs underlying hepatocellular steatosis have been investigated as potential therapeutic agents to treat NAFLD. Since the inhibition of miR-34a in a mice model improved hepatic steatosis by increasing PPARα levels promoting lipid oxidation [74], targeting miR-34a/PPARα signaling holds promise as an interesting future strategy for clinical miRNA therapeutic applications against NAFLD. Of special note, the antagomir circRNA_0046366 antagonized miR-34a and restored PPARα expression which alleviated NAFLD in an in vitro and in vivo model [75,76].

Further evidence for the involvement of miRNAs in NAFLD development can be found in one of the cell’s natural rescue mechanisms for the disease. More specifically, it has been demonstrated that the increased lipid accumulation in the liver of NAFLD patients triggers protein folding stress in the endoplasm reticulum (ER). Subsequently, more unfolded proteins accumulate in the ER leading to the activation of the unfolded protein response (UPR) [77,78,79]. The most conserved UPR pathway that has been proven to be important for NAFLD is the inositol-requiring enzyme 1α (IRE1α)/X-box binding protein 1 (XBP1) pathway [77]. IRE1α is a stress sensor activated by ER stress, which splices the mRNA of the XBP1 via its RNase activity. This spliced XBP1 will then activate the gene expression of a subset of UPR-associated regulators [80,81]. Wang et al. further showed that IRE1α is responsible for the degradation of specific miRNAs including miR-200 and miR-34. These miRNAs can target the mRNA of nuclear receptors such as PPARα mRNA as discussed above. The decrease in these miRNAs targeting PPARα mRNA by a deficiency of IRE1α leads to exacerbated hepatic steatosis in both in vivo and in vitro diet-induced NAFLD models [72]. In conclusion, miRNA regulation is strongly modulated by protein-folding stress responses during lipid homeostasis.

## 4. Epigenetic Interaction Partners in Crime in PPARα-Dependent Liver Pathologies

Besides epigenetic control mechanisms of PPARα protein expression, epigenetic enzymes can also modulate PPARα functions as direct interaction partners during NAFLD progression. Hence, the better characterization of epigenetic binding partners of PPARα may offer new therapeutic perspectives for epigenetic drugs in NAFLD treatment.

### 4.1. PPARα Interactions with Histone Modifying Enzymes

#### 4.1.1. SIRT1

Sirtuins (SIRT) are conserved NAD^+^-dependent class III histone deacetylases, highly dependent on the cellular metabolism. Hence, they are considered as cellular sensors of energy status in response to diet and environment to protect against metabolic stress. In mammals there are seven sirtuins (SIRT1-7), located in different cellular components [82,83]. Several of these sirtuins play a key regulatory role in both fasting and NAFLD conditions. First of all, the nuclear SIRT1 induces a metabolic switch during fasting conditions to restore the energy balance in the cell. Therefore, it will deacetylate several transcription factors in the liver, heart, adipocytes and skeletal muscle that induce an increase in fatty acid use and glucogenesis and other transcription factors that decrease glycolysis and fatty acid synthesis. In the mitochondria, upregulation of SIRT3 and downregulation of SIRT4 will increase fatty acid oxidation and oxidative stress during fasting [83,84]. Therefore it is not surprising that SIRT1, SIRT3 and SIRT6 have been reported to protect against fatty liver disease by controlling the expression of lipogenic enzymes, mitochondrial function and the stimulation of fatty acid oxidation, respectively [85].

**Figure 2:**
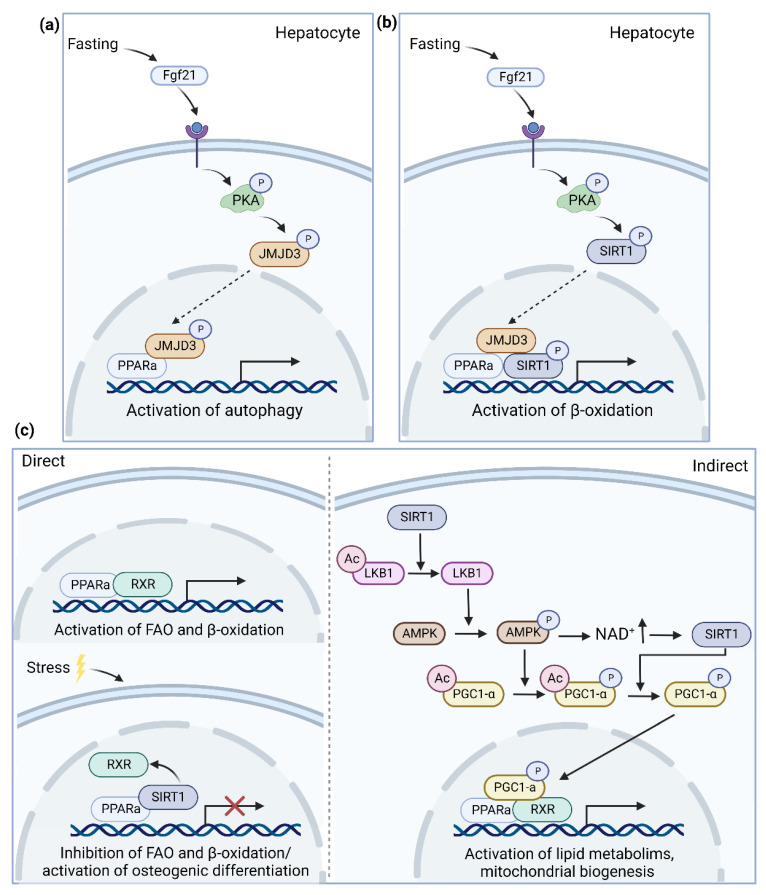
Overview of possible pathways leading to a direct interaction of PPARα with histone-modifying enzymes regulating diverse pathways. (**a**) JMJD3 can activate autophagy under starvation by interacting with PPARα (**b**) PPARα has been described to recruit both JMJD3 and SIRT1 to activate β-oxidation genes under fasting conditions (**c**) It is not clear yet if the bidirectional regulation of PPARα and SIRT1 target genes is regulated by the direct interaction between PPARα and SIRT1 or the indirect interaction via the deacetylation of PCG1-α. Abbreviations: peroxisome proliferator-activated receptor-α, PPARα; Fibroblast growth factor 21, Fgf21; Protein kinase A, PKA; Jumonji D3, JMJD3; Sirtuin 1, SIRT1; retinoid X receptor, RXR; Liver Kinase B1, LKB1; AMP-activated protein kinase, AMPK; Peroxisome proliferator-activated receptor gamma coactivator 1-alpha, PCG1-α.

Of particular interest, several research teams have demonstrated an interaction and reciprocal transcriptional crosstalk between PPARα and histone deacetylase SIRT1 (Figure 2). On one side, one of the major regulatory targets of SIRT1 is the PPARα signaling pathway. Hence, SIRT1 activity is required to activate the transcription of PPARα target genes including FGF21 in the liver [86]. Moreover, it has been reported that natural compounds and drugs used for the treatment of NAFLD targeting the PPARα signaling pathway are dependent on SIRT1 activity [87,88,89]. Moreover, in both adipocytes and hepatocytes, it has been shown that the depletion of SIRT1 reduces the expression of several PPARα target genes related to lipid metabolism and mitochondrial biogenesis [90,91]. Reciprocally, PPARα agonists including fenofibrate, WY1643 and GW7647 or the fasting-increased expression of PPARα have been reported to promote SIRT1 activity [92,93,94,95].

Whether these effects are mediated via direct interaction between PPARα and SIRT1 or require an indirect interaction via the deacetylation of Peroxisome proliferator-activated receptor gamma coactivator 1-alpha (PCG1-α) is not yet fully understood. First, the direct interaction between SIRT1 and PPARα has been shown to affect the expression of both SIRT1 and PPARα target genes depending on the cell type. Gong et al. showed with a luciferase assay in adipocytes that under high-fat conditions PPARα and SIRT1 formed a direct interaction when both genes were overexpressed [96]. This interaction induced osteogenic differentiation via the SIRT1 dependent pathway. Further, this direct interaction was also confirmed in the heart by Villarroya et al. where the interaction of PPARα and SIRT1 under a high-fat diet reduced the binding of PPARα with the RXR receptor and p65. This reduced interaction led to an upregulation of the PPARα pro-inflammatory target genes and a downregulation of fatty acid oxidation in the heart [97]. According to Oka et al., the change in the interaction partner of PPARα was due to imperfect PPAR responsive element (PPRE) binding sites that made the interaction of PPARα with the RXRα receptor unstable [98,99]. Subsequently, when PPARα is upregulated under stress conditions (i.e., heart failure or an HFD), PPARα is able to bind to other proteins, including RXR and SIRT1 [98,99,100]. The direct interaction of PPARα and SIRT1 has also been proven by coimmunoprecipitation in the liver [91]. Interestingly, in the liver, the interaction between PPARα and SIRT1 is increased when PPARα is activated and abolished when PPARα is poly(ADP-ribosyl)ation by PARP1 [101]. Since PPARα and SIRT1 are downregulated and PARP1 is upregulated in NAFLD patients, the study of this interaction is of high importance for the treatment of the disease [17,101,102].

Besides its direct interaction, SIRT1 also indirectly activates PPARα functions via the AMPK-Sirt1-Pgc-1α signaling pathway. AMPK and SIRT1 are both metabolic energy sensors that form a positive feedback loop to finetune the cellular energy metabolism status [103]. More specifically, AMPK can be activated by SIRT1 through the deacetylation of liver Kinase B1 (LKB1), while SIRT1 is activated by AMPK through the synthesis of NAD^+^ [104,105]. Subsequently, activated SIRT1 can deacetylate and activate PCG1-α, while AMPK enhances its activity by phosphorylation [91,106,107]. This deacetylated PCG1-α has been reported to function as a coactivator of PPARα, leading to the activation of several PPARα target genes involved in the lipid metabolism, mitochondrial biogenesis and (anti)-inflammatory pathways [106,108,109,110,111]. All these pathways have been described for their role in the development and progression of metabolic diseases, indicating the importance of this pathway for the treatment of several metabolic diseases including diabetes and NAFLD [112,113,114].

#### 4.1.2. JMJD3

JMJD3 is a histone lysine demethylase that belongs to the KDM6 family and epigenetically activates genes by demethylating the repressive histone H3K27-me3 mark [115]. It has an established role in development, differentiation, immunity and extending the lifespan in response to mild mitochondrial stress [115,116]. However, recently JMJD3 has also proven its role in the initiation of autophagy and metabolic regulation by the interaction with PPARα [117,118] (Figure 2).

Autophagy is an essential catabolic process for cellular survival and energy homeostasis under nutrient deprivation [119,120]. It recycles cytoplasmatic components (e.g., organelles) to new building blocks (e.g., amino acids) for cellular renovation and provides free fatty acids for β-oxidation by degrading intracellular lipid stores for energy production [120]. Byun et al. first reported a role of JMJD3 in the activation of autophagy under starvation by interacting with PPARα. Upon fasting, FGF21 (Fibroblast growth factor 21) signaling is activated which induces the phosphorylation of JMJD3 at Thr-1044 by PKA (Protein kinase A). This leads to the activation of JMJD3, increasing its nuclear localization and interaction with PPARα to transcriptionally activate autophagy [117]. Dysregulation of autophagy has been linked to several diseases, including NAFLD [121]. Moreover, the expression of both JMJD3 and PPARα is decreased in NAFLD patients [17,117]. However, to determine whether there is a causal link between the FGF21-JMJD3-PPARα axis leading to a decreased expression of autophagy genes and the development of NAFLD, further investigation is necessary. In addition to autophagy, an interaction between JMJD3, PPARα and SIRT1 also activates mitochondrial fatty acid β-oxidation [118]. Under fasting conditions, PPARα recruits both JMJD3 and SIRT1 to activate β-oxidation genes. Next, SIRT1 is phosphorylated at Ser434 upon PKA activation, inducing the formation of the JMJD3–SIRT1–PPARα complex at PPRE of β-oxidation network genes. This interaction is abolished when one of the genes is downregulated, indicating a strong positive autoregulatory loop. Moreover, liver specific downregulation of JMJD3 impaired mitochondrial β-oxidation, liver steatosis and glucose and insulin intolerance in mice fed a normal chow diet [118].

Both studies indicate an interesting link between the epigenetic enzyme JMJD3 and PPARα at the crossroad of autophagy and β-oxidation in NAFLD, which could be targeted by epigenetic drugs.

### 4.2. PPARα Interactions with DNA-Modifying Enzymes

#### 4.2.1. TET Enzymes

**Figure 3:**
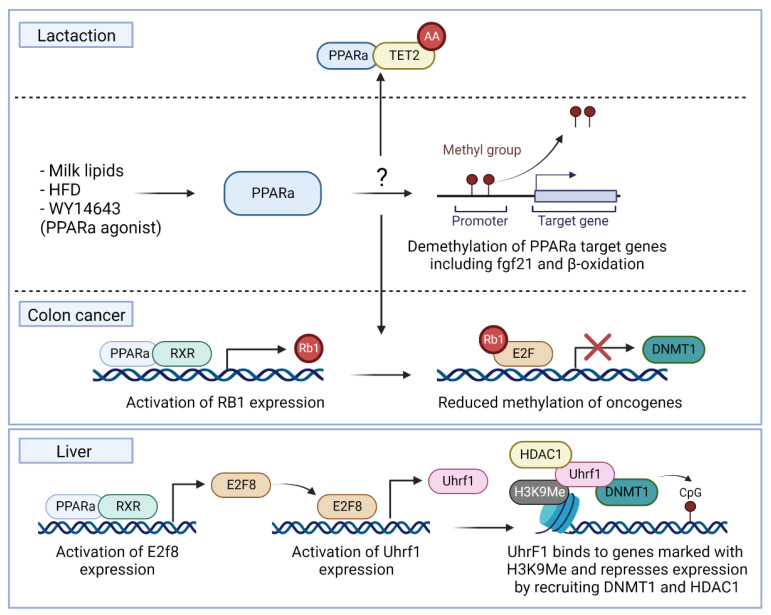
Overview of PPARα as an epigenetic regulatory driver regulating the expression of its target genes by interacting with DNA-modifying enzymes. Possible pathways for demethylation could be a direct interaction with TET2 as found during lactation or by inhibiting DNMT1 via the RB1/E2F pathway due to the activation of Rb1 expression by PPARα as found in colon cancer. Moreover, PPARα can activate the expression of the epigenetic regulator Uhrf1 by inducing the expression of the E2f8 transcription factor. Uhrf1 is known to recruit DNMT1 to target genes with a H3K9me3 histone mark leading to hypermethylation and downregulation. Abbreviations: peroxisome proliferator-activated receptor-α, PPARα; tet methylcytosine dioxygenase 2, TET2; Fibroblast growth factor 21, Fgf21; High-fat diet, HFD; retinoid X receptor, RXR; retinoblastoma tumor suppressor gene, RB1; E2F transcription factor, E2F; DNA methyltransferase I, DNMT1; E2F Transcription Factor 8, E2f8; Ubiquitin-like with PHD And Ring Finger Domains 1, Uhrf1; Histone deacetylase 1, HDAC1.

Pang et al. observed an association of decreased expression of TET1 and TET2 with increased methylation of PPARα in the mice embryos of mothers fed an HFD during gestation [122]. Reciprocally, PPARα activation induced demethylation of its target genes, including fgf21 and several genes of the β-oxidation both during the perinatal period induced by milk lipids or in adolescent rats induced by an HFD [123,124,125,126]. Moreover, mouse livers of mice treated with the PPARα agonist WY-14643 showed a demethylation of the growth arrest DNA damage-inducible beta (GADD45b) gene [127]. Although it is uncertain how PPARα induces this demethylation, Yuan et al. reported an increased expression of TET2 and TET3 during lactation together with a possible interaction of PPARα with the TET2 enzyme [124] (Figure 3). Moreover, it has been shown that ascorbic acid, a cofactor for TET enzymes, is necessary to induce the proper demethylation of PPARα target genes, including fgf21, in offspring [128].

#### 4.2.2. DNMT Enzymes

Besides interactions of PPARα and TET enzymes in early development, associations of PPARα inhibition with the increased expression of DNA methyltransferase I (DNMT1) and protein arginine methyltransferase 6 (PRMT6) have already been demonstrated in colon cancer and the liver [129,130]. More specifically, Luo et al. reported that in colon cancer, due to the downregulation of PPARα, less RB1 protein will be expressed. Considering the repressive role of RB1 on E2F transactivation and the E2F binding sites in the *DNMT1* and *PRMT6* promoters, this will induce an upregulation of DNMT1 and PRMT6 [129,131]. This upregulation will further lead to the decreased expression of tumor suppressor genes and the development of more severe colon cancer [129] (Figure 3). This inhibitory role of PPARα on DNMT1 has also been confirmed by Kong et al. showing an inhibition of DNMT1 followed by an activation of the tumor-suppressor gene CDKN2A due to lower methylation, induced by a treatment with the PPARα agonist fenofibrate [130]. Besides, Aibara et al. reported that PPARα activation in the liver causes hepatocyte proliferation by the activation of DNMT1 via the expression of another E2F transcription factor called E2f8. This E2f8 transcription factor is known to induce the expression of the epigenetic regulator Uhrf1, which binds to target genes with a H3K9me3 histone mark and recruits DNMT1 and HDAC1 to regulate expression [132]. These studies established an interesting functional epigenetic regulatory driver role of PPARα to epigenetically regulate targets by activating transcription factors regulating the activity of DNMT1 (Figure 3). Moreover, Hervouet et al. demonstrated a direct interaction of PPARα with DNMT1 [133]. In mice, a high-fat diet-induced NAFLD phenotype was also accompanied by a decreased expression of DNMT3a and DNMT3b [134]. Further molecular characterization of possible interactions of PPARα with DNMTs and TET enzymes may reveal new therapeutic targets for epigenetic drugs against NAFLD.

## 5. Conclusions and Future Perspectives

Since the discovery of PPARα in 1990, this nuclear receptor has been known as a master regulator of the metabolism because of its regulatory role in the lipid metabolism [135]. Therefore, it has been an attractive therapeutic target in the research for a therapy of NAFLD. However, although various epigenetic enzyme interaction partners (SIRT1, JMJD3, TET, DNMT1) have already been identified for PPARα, there remains a research gap which addresses the role of PPARα as an epigenetic driver in NAFLD progression. As summarized in this review, some clear associations and interactions of PPARα with epigenetic modifying enzymes are involved in the metabolism. However, the mechanistic pathways behind these associations are incomplete and need further research. This is important since epigenetic modifications are reversible and dynamic during the development and progression of NAFLD; therefore, combination therapies of epigenetic drugs with currently investigated PPAR agonists–antagonists hold promise for future drug discovery pipelines against NAFLD. Since there is still no FDA-approved therapy for NAFLD, many drugs that are under investigation include PPARα agonists. The first type of agonists tested were fibrates, which showed promising results in preclinical trials. Although the effects on lipid metabolism showed beneficial effects for the development of NAFLD-related cardiovascular disease including atherosclerosis, the liver-related beneficial outcomes were not translated in the clinical trials with NALFD and NASH patients [136,137,138,139,140] (reviewed in [18]). Another PPARα agonist called Pemafibrate has been approved and marketed in Japan for the treatment of dyslipidemia [141,142]. Although this drug has shown promising results based on blood-based markers of NAFLD (e.g., ALT, AST, TG), histological liver outcomes are missing [18,143]. Therefore, it needs further investigation for the treatment of NAFLD patients in clinical trials. Dual or pan PPAR agonists have shown more promising results as potential treatments, especially the pan PPAR agonist lanifibranor, which is currently being further investigated in a phase III clinical trial with NASH patients [18,144]. Moreover, the dual PPARα and PPARγ agonist pioglitazone, which is part of the thiazolidinediones, has also shown histological and metabolic improvements in NASH patients. However, large clinical trials are necessary to assess its long-term efficacy and to evaluate safety towards heart failure [145,146,147]. Besides targeting multiple PPAR isoforms, it could also be interesting in the future to combine PPARα agonists with epigenetic drugs. For example, currently investigated epigenetic modulator compounds vitamin E and resveratrol, which inhibit DNMT1 expression and activate SIRT1 respectively, may also affect the epigenetic regulation of PPARα in NAFLD as shown in this review [148,149]. Therefore, a better functional molecular characterization of the epigenetic interaction partners of PPARα may provide novel mechanistic insights for innovative therapeutic targeting strategies which could restore lipid energy homeostasis and ameliorate NAFLD.

## Data Availability

The data that support the findings of this study are available from the corresponding author upon reasonable request.
